# 

**Published:** 1907-05

**Authors:** 


### CONTENTS.

ORIGINAL COMMUNICATIONS—
Special Announcement .......... 65
The Doctor in Relation to Exercise as a Factor in Medicine and Surgery. By Theodore Toepel, M. D. Atlanta, Ga .......... 67
Discussion of Dr. Toepel's Paper .......... 73
Treatment of Children's Mouths. By Geo. S. Tignor, A. B., D. D. S., Atlanta, Ga .......... 74
Discussion os Dr. Tignor's Paper .......... 77
Methylene Blue in the Treatment of Cancer. By Henry R. Slack, Ph. M. D., President of Georgia Pasteur Institute, Atlanta, Ga.; Superintendent LaGrange Sanatorium, LaGrange, Ga. .......... 79
Some Personal Observations in the Fitting of Hernia-Supports. By Arnold H. Lindorme, M. D., Atlanta .......... 83
EDITORIAL NOTES AND COMMENTS—
The XVI International Medical Congress at Budapest in 1909 .......... 87
The Conncil on Pharmacy and Chemistry .......... 88
The Management of Convalescence .......... 90
NEWS AND NOTES .......... 92
Death of Theodore D. Buhl .......... 95
BOOK REVIEWS .......... 97
SELECTIONS AND ABSTRACTS—
Olive Oil in Cholelithiasis .......... 100
Dr. Edw. Register's New Book .......... 100
Buttermilk as an Infant Food .......... 101
Rheumatism and Its Treatment .......... 102
Pruritus Ani .......... 102
Cesarean Section Performed by a Cow .......... 103
The Danger of Dust as a Cause of Tuberculosis .......... 103
Emasculation for Criminal Assaults and Incest .......... 105
Forms of Albumen in the Urine .......... 104
Success .......... 105
Styes .......... 106
The Theory of the Toxic origin of Pernicious Anemia .......... 107
The State and Tuberculosis .......... 107
Dedication of the Barlow Library of Los Angeles .......... 108
The Dissemination of Intra-Abdominal Malignant Disease by Means of the Lymphatics and Theoracic Duct .......... 109
A Contribution to the Histo-Pathology and the Theory of Drug Eruptions .......... 110
The Use of Digitalis in Valvular Disease of the Heart .......... 112
Pneumonia .......... 113
Hydrotherapy in Tuberculosis .......... 114
Heedlessness in the Seleetion of Officers of Medical Societies .......... 115
Tuberculosis of Joints .......... 117
The Relation of Chlorids to Edema .......... 120
The Diagnosis of Scarlet Fever. By A. R. Braunlich, M. D .......... 123
Surgical Suggestions .......... 127

